# The genetic mapping and phenotypic analysis of *Patronin ^F.1.1^* in *Drosophila melanogaster*

**DOI:** 10.17912/micropub.biology.001564

**Published:** 2025-06-09

**Authors:** Lucas Gruber, Idalia Soto, Tristan Correa, Esmeralda Apodaca, Brian Arreola, Jhamiley Cabral, Kristine Chen, Miguel Correo Galicia, Joanna Gomez, Ellaine Hao, Brisa Hernandez, Darien L Holland, Crystal Jara-Pichardo, Gwyniever M Lonzame, Abigail Ortiz Olguin, Rheana Romero, Devon Walker, Spencer G Amacher, Cooper Christie, Lillian G Coats, Aubrey Gerhardt, Claire A Holsted, Stephen R Kraizel, Ashley Krumlaw, Alex Stevens, Haleigh Stover, Chloe M Sullivan, Hailey Wyse, Danielle R Hamill, Jacob D Kagey, Kayla L Bieser

**Affiliations:** 1 Nevada State University, Henderson, Nevada, United States; 2 Ohio Wesleyan University, Delaware, Ohio, United States; 3 University of Detroit Mercy, Detroit, Michigan, United States

## Abstract

The multi-institutional Fly-CURE project is an undergraduate genetics research initiative centered on *Drosophila melanogaster* as a model organism. This study aimed to characterize and map mutations discovered through a Flp/FRT EMS screen to investigate complex interactions among genes associated with cell division, growth, and apoptosis leading to abnormal cell proliferation. The *F.1.1* mosaic phenotype resulted in a rough eye phenotype with an overall decrease in representation of mutant tissue. To genetically map the location of the *F.1.1* mutation, flies with genotype *FRT42D,F.1.1, Dark ^82^ /CyO* were crossed with the Bloomington 2R Deficiency Kit. The resultant F1 progeny were analyzed to pinpoint mapping deficiencies. The genomic region containing the *Patronin* gene was identified and sequencing confirmed the novel allele of *Patronin ^F.1.1^* .

**Figure 1. Phenotypic characterization, genetic mapping, and molecular analysis of the F.1.1 mutation:**
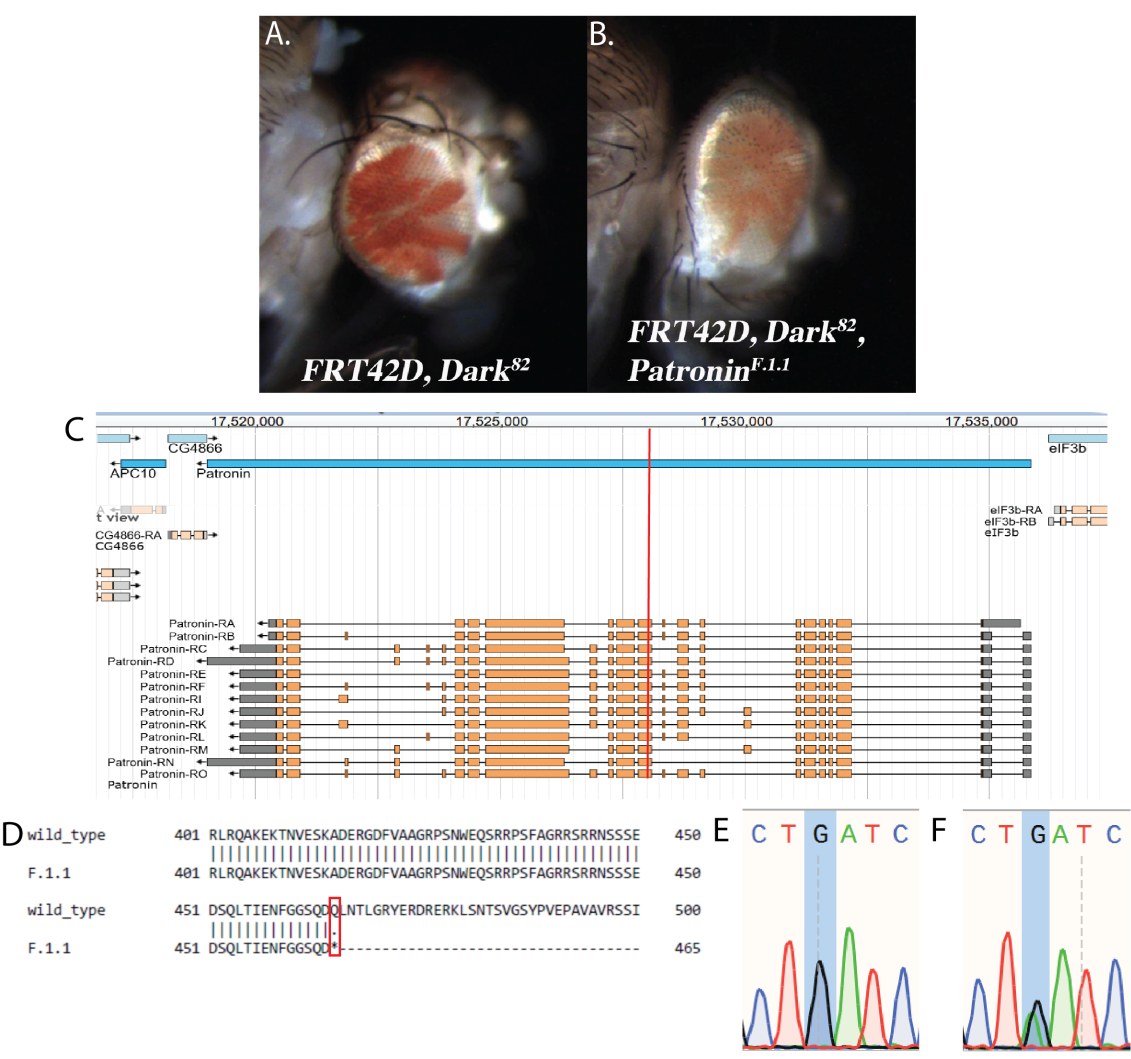
(A) Control adult mosaic eye phenotype of *FRT42D, Dark ^82^* displaying a Red>White ratio of pigmentation. (B) Adult mosaic eye of *FRT42D, Dark ^82^ , Patronin ^F.1.1^* displayed a decrease in red pigmentation (mutant tissue) with abnormal patterning and eye development (rough eye). (C) The *F.1.1* fly failed to complement *Patronin* . The genomic view of the *Patronin* locus (FlyBase) showing the *Patronin* transcripts and location of premature stop codon (red line) *.* (D) Alignment of the wild-type and *F.1.1 Patronin* amino acid sequences, showing a stop codon in the *F.1.1* mutant (p.Gln465*). (E) Segment of DNA sequencing chromatogram of the *FRT42D, Dark ^82^* control and (F) the *Patronin* single nucleotide substitution in *F.1.1* (G to A at 2R:17,528,031).

## Description

An ethyl methanesulfonate (EMS) genetic screen was conducted in *Drosophila melanogaster* to isolate genetic mutations involved in the regulation of cell growth, cell division, and development (Kagey et al. 2012). EMS mutagenesis was done in a *FRT42D, Dark ^82^* background which was utilized to block apoptosis as *Dark ^82^* is a null allele of the *Death-associated APAF-1 killer* ( *Dark* ) on the distal arm of chromosome 2R (Akedemir et al. 2006, Mills et al. 2006). The *Dark ^82^* mutation sensitizes the strain and may allow for isolation of overgrowth mutants that would be missed if apoptosis were not also blocked. This loss-of-function mutation also contains a *mini-white ^+^* gene insertion allowing for the identification of homozygous mutant tissue in the eye based on observable red pigmentation after mitotic recombination. The FLP/FRT mitotic recombination system was used allowing for expression of the mutant phenotype in specific areas of the flies (e.g. the eyes) and gets around the limitation that mutations in genes that control cell growth and division often cause embryonic lethality (Xu and Rubin 1993). Here we describe the phenotypic analysis and genetic mapping of the *F.1.1* mutation, previously isolated from the genetic screen. This analysis was carried out by 2 independent groups of undergraduate researchers at Nevada State University and Ohio Wesleyan University participating in the course-based undergraduate research experience, Fly-CURE (Merkle et al. 2023).

To visualize the mutant phenotype of *F.1.1* , males of both control ( *FRT42D, Dark ^82^ /CyO* ) and mutant ( *FRT42D, Dark ^82^ , F.1.1/CyO* ) flies were crossed to virgin female *FRT42D;Ey>Flp* flies generating mosaic eyes of homozygous wild type and homozygous mutant clones (Kagey et al. 2012). The control cross displayed a 60:40 red>white phenotypic ratio whereas the mutant cross had a reduction in red pigmentation (mutant cells) and a rough eye appearance ( Fig. 1A and 1B, respectively).

To narrow down the location of the F.1.1 mutation on chromosome 2R, genetic complementation mapping was done by crossing *FRT42D, F.1.1, Dark ^82^ /CyO* females with males of the BDSC Df(2R) kit (Cook et al. 2012). Complementation was assessed by observing the wing phenotypes of the F1 progeny; the presence of both curly and straight wings indicated complementation. Failure to complement (FTC) was indicated by ≥100 F1 progeny scored with an absence of straight wings due to homozygous lethality in the mutated gene. In the first round of mapping, *F.1.1* failed to complement deficiency line *Df(2R)BSC355* within the BDSC 2R deficiency kit. Further mapping was conducted with smaller deficiencies within this region and it was found that complementation occurred with deficiency lines *Df(2R)BSC406, Df(2R)BSC161,* and *Df(2R)Exel7149* . These results indicated the smallest region that failed to complement was between 2R:17,518,127..17,536,673 for which there were two genes present, *Patronin* and *elF3b* (Table 1). Additional complementation mapping resulted in a failure to complement two *Patronin* alleles ( *Patronin ^k07433^* and *Patronin ^EY05252^* ) indicating that *Patronin* is responsible for the *F.1.1* phenotype, identifying a novel allele of *Patronin , Patronin ^F.1.1^* . In contrast, *Patronin ^F.1.1^* complemented in crosses with two alleles of *elF3b* (Table 1).

DNA was purified from control ( *FRT42D, Dark ^82^ /CyO* ) and mutant ( *FRT42D, Dark ^82^ , Patronin ^F.1.1^ /CyO* ) *Drosophila melanogaster* stocks. PCR primers were designed to allow amplification of the *Patronin* gene from control and mutant DNA. Sanger sequencing was conducted by the UNLV Genomics Core Facility. Following sequencing, data analysis was performed by students using SnapGene Viewer. A single nucleotide alteration at 2R:17,528,031 was identified where a guanine was replaced with an adenine demonstrating heterozygosity ( Fig. 1F ) as compared to the single guanine peak in the control ( Fig. 1E ). This alteration led to a premature stop codon affecting all known isoforms produced by *Patronin* ( Fig. 1C and 1D). Protein alignment between the wild type and mutant sequences ( Fig. 1D ) revealed that translation stops at amino acid residue 465. We used FlyBase (release FB2024_04) to determine this premature stop codon results in the deletion of 1,165 residues in Patronin's isoform C. Contained within these lost residues are the CAMSAP_CH, CAMSAP_CC1, tolA_full, and CAMSAP_CKK domains which are conserved regions associated with microtubule and spectrin binding (Öztürk-Çolak et al. 2024).

*Patronin* is a microtubule (MT) minus-end binding protein that stabilizes microtubules by preventing depolymerization at their minus-ends which is vital for spindle elongation during cell division (Pavlova et al. 2019). Its mitotic roles may include control over anaphase B in embryonic cells and central spindle assembly during the asymmetric division of sensory organ precursor cells (Pavlova et al. 2019). Non-mitotic functions of *Patronin* include stabilizing polarized non-centrosomal MT arrays in the oocyte (Pavlova et al. 2019). Current studies into the *Patronin* gene suggest that it shares homologous sequence identity and function with human *CAMSAP* (calmodulin regulated spectrin associated proteins) genes which interact with MTs and participate in mitotic and non-mitotic roles (Pavlova et al. 2019). In mammals, there are three CAMSAP proteins: CAMSAP1, CAMSAP2, and CAMSAP3. CAMSAP1 is limited to the mitotic spindles, and its loss slightly reduces spindle length. CAMSAP2 and CAMSAP3 stabilize the minus ends of non-centrosomal microtubules at adherens junctions (Pavlova et al. 2019). CAMSAP1 mutations serve as a predictive indicator in cancer patients triggering anti-tumor immunity, mediate tumor cell apoptosis, improve prognosis, and improve sensitivity to platinum-based chemotherapy (Yi et al. 2022). In *Drosophila,* it has been demonstrated that in the absence of *Patronin* , there is microtubule disorganization resulting in short spindles during mitosis (Goodwin and Vale 2010). The reduction in pigmentation visualized in *Patronin ^F.1.1^* , is likely the result of this microtubule disorganization and therefore decreased cellular proliferation.

## Reagents

**Table 1: Complementation tests conducted with mutant *F.1.1***

**Table:** 

**Bloomington 2R Deficiencies Failing to Complement**			
**Deficiency**	**BDSC Stock #**	**Region**	**Complementation Result**
*Df(2R)BSC161*	BDSC_9596	2R: 17304783..17484828	Complements
*Df(2R)BSC355*	BDSC_24379	2R: 17462347..17536673	Fails to complement
*Df(2R)BSC406*	BDSC_24430	2R: 17462347..17518127	Complements
*Df(2R)Exel7149*	BDSC_7890	2R: 17581496..17691787	Complements
** Single Genes Tested Within Mutated *F.1.1* Region **			
**Gene**	**BDSC Stock #**	**Allele**	**Complementation Result**
*Patronin*	BDSC_10672	* Patronin ^k07433^*	Fails to complement
*Patronin*	BDSC_16647	* Patronin ^EY05252^*	Fails to complement
*elF3b*	BDSC_85705	* elF3b ^f05638^*	Complements
*elF3b*	BDSC_20931	* elF3b ^EY14430^*	Complements

*w; FRT42D, Dark ^82^ /CyO* (Akdemir et al. 2006)

*w; FRT42D, Patronin ^F.1.1^ , Dark ^82^ /CyO* (this manuscript)

*y w; FRT42D; ey-FLP* ( BDSC 8211 )

Bloomington *Drosophila* Stock Center 2R Deficiency Kit (Cook et al. 2012)
